# High prevalence of multidrug-resistant Gram-negative bacteria carriage in children screened prospectively for multidrug resistant organisms at admission to a paediatric hospital, Hamburg, Germany, September 2018 to May 2019

**DOI:** 10.2807/1560-7917.ES.2022.27.15.2001567

**Published:** 2022-04-14

**Authors:** Safiullah Najem, Dorothée Eick, Johannes Boettcher, Annette Aigner, Mona Aboutara, Ines Fenner, Konrad Reinshagen, Ingo Koenigs

**Affiliations:** 1 Department of Paediatric Surgery, Altona Children's Hospital, Hamburg, Germany; 2 Department of Paediatric Surgery, University Medical Center Hamburg-Eppendorf (UKE), Hamburg, Germany; 3 Department of Child and Adolescent Psychiatry and Psychotherapy, University Medical Center Hamburg-Eppendorf, Hamburg, Germany; 4 Institute of Biometry and Clinical Epidemiology, Charité – Universitaetsmedizin Berlin, Berlin, Germany; 5 Institute of Medical Biometry and Epidemiology, University Medical Center Hamburg-Eppendorf, Hamburg, Germany; 6 Department of General Paediatrics, Altona Children's Hospital, Hamburg, Germany; 7 Laboratory Dr. Fenner and colleagues, Hamburg, Germany

**Keywords:** multi-drug-resistant organisms, prevalence, colonization, childhood, risk factors

## Abstract

**Background:**

Increasing resistance to antibiotics poses medical challenges worldwide. Prospective data on carriage prevalence of multidrug resistant organisms (MDRO) in children at hospital admission are limited and associated risk factors are poorly defined.

**Aim:**

To determine prevalence of MDRO carriage in children at admission to our paediatric hospital in Hamburg and to identify MDRO carriage risk factors.

**Methods:**

We prospectively obtained and cultured nasal/throat and inguinal/anal swabs from children (≤ 18 years) at admission between September 2018 and May 2019 to determine prevalence of meticillin-resistant *Staphylococcus aureus* (MRSA), multidrug-resistant Gram-negative bacteria (MRGN) and vancomycin-resistant enterococcus (VRE) and associated species. We collected medical histories using a questionnaire and evaluated 31 risk factors using logistic regression models.

**Results:**

MDRO carriage prevalence of 3,964 children was 4.31% (95% confidence interval (CI): 3.69–5.00). MRSA carriage prevalence was 0.68% (95% CI: 0.44–0.99), MRGN prevalence was 3.64% (95% CI: 3.07–4.28) and VRE prevalence 0.08% (95% CI: 0.02–0.22). MDRO carriage was associated with MRGN history (odds ratio (OR): 6.53; 95% CI: 2.58–16.13), chronic condition requiring permanent care (OR: 2.67; 95% CI: 1.07–6.13), antibiotic therapy (OR: 1.92, 95% CI: 1.24–2.94), living in a care facility (OR: 3.34; 95% CI: 0.72–12.44) and refugee status in previous 12 months (OR: 1.91; 95% CI: 0.27–8.02). Compared to established practice, screening using risk-factors had better diagnostic sensitivity (86.13%; 95% CI: 80.89–91.40) and specificity (73.54%; 95% CI: 72.12–74.97).

**Conclusion:**

MRGN carriage was higher than MRSA and VRE. Extended risk-factor-based admission screening system seems warranted.

## Introduction

Worldwide, multidrug-resistant organisms (MDRO) are an increasing medical and economic burden [1]. In particular, the increasing antibiotic resistance in Gram-negative bacteria poses major challenges to medical care [2,3]. Although meticillin-resistant *Staphylococcus aureus* (MRSA) colonisation shows a decreasing prevalence, the occurrence of multidrug resistant Gram-negative bacteria (MRGN), such as extended spectrum beta-lactamase (ESBL) *Escherichia coli* and *Klebsiella pneumoniae*, is increasing [2].

Different international prevalence estimates and risk factors for MDRO result in different strategies for MDRO management [4-8]. For example, our hospital isolates patients only after receiving a positive test result for MDRO, whereas other countries isolate newly admitted patients until a negative test result is received. While standardised MRSA screening is established in most European hospitals, the focus has changed to MRGN screening in adult patients in recent years [2,6,9-11]. Although recent studies published between 2013 and 2018, from Europe and the United States (US) have reported transmission routes for MRGN colonisation, such as direct person-to-person transmission, indirect transmission through contaminated areas or materials (e.g., kitchen towels), antibiotic use, contact with healthcare facilities in high MDRO prevalence countries and travel history to Africa and Asia, most risk factors seem unknown [2,6,12-14]. Because of belated or incomplete access to professional medical history at admission, MDRO detection is often delayed, which can increase morbidity and mortality [1].

European studies have shown an overall MRSA prevalence between 1.3% [7] and 2.0% [15], and a MRSA prevalence in children between 1.2% [15] and 2.7% (95% CI: 2.2–3.1) [16]. A meta-analysis conducted by Gesualdo et al. in 2013, ascertained a MRSA prevalence of 5.2% (95% CI: 3.1–7.3) in children with underlying medical conditions and chronic diseases, of 2.3% (95% CI: 1.8–2.7) in healthy children, and of 5.4% (95% CI: 3.1–7.7) in hospitalised children, which is higher than the 3.0% (95% CI: 2.4–3.6) in the general paediatric population [16]. According to a nationwide one-day point prevalence study in German hospitals focusing on adult care in 2014, MRSA prevalence was 1.64% (95% CI: 1.46–1.82). MRGN prevalence was 1.65% and of these were 1.4% 3MRGN and 0.25% 4MRGN. 3MRGN *E. coli* prevalence was 0.75% (95% CI: 0.60–0.89), *Klebsiella* spp. prevalence was 0.22% (95% CI: 0.15–0.29), *Pseudomonas* spp. prevalence was 0.21% (95% CI: 0.10–0.31), *Enterobacter* spp. prevalence was 0.10% (95% CI: 0.05–0.15), *Acinetobacter* spp. prevalence was 0.02% (95% CI: 0.00–0.03) and other non-specified 3MRGN prevalence was 0.10% (95% CI: 0.05–0.15). 4MRGN *Pseudomonas* spp. prevalence was 0.17% (95% CI:0.06–0.29), 4MRGN *Klebsiella* spp. prevalence was 0.04% (95% CI: 0.00–0.07) and other 4MRGN ranged between 0.01% and 0.04%. Vancomycin-resistant enterococcus (VRE) prevalence was 0.25% (95% CI: 0.13–0.37) [9]. Data published from 2013 from German hospitals reveal an increase in third-generation cephalosporin-resistant Enterobacteriaceae colonisation of up to 9.5%, and European studies show an increasing high ESBL carriage of up to 5.0% (95% CI: 3.4–6.6) [2] in adults [10,14,17-19].

With respect to MDRO prevalence, prospective data are scarce for children and adolescents [20]. In addition, changes in prevalence of MRSA and MRGN have not been adequately investigated. Furthermore, no uniform screening standards for MDRO detection in children have been established [6].

This study aimed to investigate the prevalence of MDRO carriage (MRSA, MRGN and VRE) at admission to the Altona Children’s Hospital (AKK) in Hamburg, Germany for scheduled or emergency hospital admissions as well as to determine clinical, epidemiological and microbiological risk factors associated with MDRO carriage. This information was used to critically evaluate the current MDRO screening at admission.

## Methods

### Study design

This prospective observational study was performed at one hospital. After written informed consent was obtained, participants were recruited over 9 months, between September 2018 and May 2019, at the AKK. With about 12,000 in-patient visits per year, the AKK is one of the largest paediatric care facilities in Germany, attracting patients from Hamburg and surrounding areas as well as from across Germany. The study included all in-patients (≤ 18 years, hereafter referred to as children) with scheduled or emergency admission from every department: general paediatrics, intensive care unit (ICU), neonatology, neurosurgery, otorhinolaryngology, orthopaedics, surgery, trauma surgery, urology, and Lufthafen, a special unit for children requiring permanent ventilation. Patients were excluded from the study if they were re-admitted to our clinic after initial recruitment.

### Data and clinical sample collection

Pooled smear tests were taken from typical regions (nasal/throat and inguinal/anal) to determine MDRO occurrence (MRSA, MRGN and VRE) and associated species. In general, the samples were taken at admission. In special emergency situations, samples were taken after the patient was in a stable condition. Additionally, we collected clinical, epidemiological and microbiological data using a questionnaire for parents and attending physicians, which was completed during admission. The questionnaire was available in Arabic, English, French, German, Persian (Farsi), Russian and Turkish to ensure inclusion of different ethnicities. Based on recommendations from the German Commission for Hospital Hygiene and Infection Prevention at the Robert Koch Institute (KRINKO) and on questionnaires from previous European studies [6,13-16,21], we evaluated six general demographic factors: (i) sex, (ii) age, (iii) department, (iv) emergency, (v) transfer (patient transferred from another hospital) and (vi) country of birth of the child and parents, and 25 specific factors (see Supplementary Tables S1–3). The patient questionnaire already in use and evaluating 17 risk factors was modified by adding eight additional risk factors: (i) history of VRE, (ii) contact with a family member treated at ICU for more than 7 days the previous 12 months, (iii) having a pet, (iv) being breastfed within the previous 12 months, (v) major surgery the previous 12 months, (vi) urogenital anomalies or recurrent urological infections, (vii) family member working in healthcare facilities with regular patient contact and (viii) stay at a neonatology unit for more than 7 days within the previous 12 months. For some questions (11 of 25), if the answer was affirmative, one to four sub-questions needed to be answered (a total of 18 sub-questions) – e.g., duration of treatment (see Supplementary Tables S1–3; 1.1, 2.1, 3.1, 4.1–2, 9.1–3, 17.1–2, 18.1, 20.1–4, 21.1, 22.1 and 24.1). Parents and attending physicians needed approximately 3 to 5 minutes to complete the whole questionnaire. Some information was derived from the medical history recorded during admission, including information from the physical examination.

### Laboratory procedures

Pooled smear tests were taken using the eSwabÔ (Copan, Brescia, Italy) collection and transport device consisting of Nylon Flocked Swabs and 1ml of Liquid Amies. Most of the smears were taken by the nursing staff and rarely by doctors. One swab was used for sampling the throat and then the nasal cavity, and another swab was used for sampling the inguinal and then the anal region. The swabs were immersed in 1mL of Liquid Amies (eSwabÔ Copan, Brescia, Italy) until processed. Smears were analysed in the same diagnostic laboratory for the entire study period. In the laboratory, nasal/throat swabs were incubated on culture media for MRSA (MRSA Chromagar, Biomerieux, Nuertingen, Germany) and ESBL (ESBL Chromagar, Biomerieux). Inguinal/anal swabs were incubated on culture media for MRSA (MRSA Chromagar, Biomerieux), ESBL (ESBL Chromagar, Biomerieux) and VRE (VRE Chromagar, MAST Diagnostica, Reinfeld, Germany). All swabs were incubated for 48 h at 36 °C. Initial observations were made after 24 h. Growing cultures were isolated and selectively differentiated. The bacteria were identified by Matrix-Assisted Laser Desorption/Ionization Time-of-Flight mass-spectrometer (MALDI-TOF Biotyper Systems, Bruker, US). Antimicrobial susceptibility testing was performed either by the disk diffusion method or by breakpoints in an automated system (Walkaway, Beckman Coulter, US), according to current European Committee on Antimicrobial Susceptibility Testing (EUCAST) susceptibility testing criteria. If an MDRO was isolated, the results were classified according to KRINKO recommendations: 2MRGN (resistant against piperacillin and cefotaxim and/or ceftazidim), 3MRGN (resistant against piperacillin, cefotaxim and/or ceftazidim and ciprofloxacin), or 4MRGN (3MRGN plus resistant against imipenem and/or meropenem) [22].

### Statistical analysis

In 2013, Wegner et al. found an MDRO (MRSA, MRGN and VRE) prevalence of 3.04% in adults [23], and a slightly lower prevalence was expected in children [5,8,15]. Sample size estimation showed that based on 4,000 patients, a prevalence of at least 2.34% could be estimated – based on a 5% significance level and the conservative assumption that precision is at one-fifth of the expected prevalence [24,25]. Hence more than 4,000 patients were recruited (4,092).

The primary endpoint was MDRO carriage prevalence at admission to the AKK, and the secondary endpoint was the prevalence of individual pathogens (MRSA, MRGN and VRE). The 95% confidence intervals (CI) for these prevalence estimates were derived based on Clopper and Pearson [26]. To visualise the trend in prevalence over age groups, a locally weighted scatterplot smoothing (LOESS) estimate was used. Associations between individual risk factors and the primary endpoint, MDRO, as well as with the secondary endpoint, MRGN, were assessed with two multivariable logistic regression models. From these, odds ratio (OR) estimates along with 95% CIs were derived. Including all potential risk factors in the model resulted in the estimation of the direct effects of each of them, independent of the other factors. As analyses were based on all available cases, 3,851 (97.1%) observations contributed to the prevalence estimate of the primary and secondary endpoint. Sensitivity and specificity of the original screening survey were assessed as in the extended version and were reported along with 95% Wald CIs.

Data collection and processing based on questionnaires and microbiological results were performed using EpiData (EpiData Association, Odense, Denmark) [27]. Statistical analyses were performed using IBM SPSS Statistics 26 (IBM, Armonk, New York, US) and R (R-Foundation, Vienna, Austria) [28,29].

## Results

### Characteristics of the study population

During the study period, 4,092 children were recruited, but 124 patients had to be excluded as they were recruited twice during the study period and four additional children were excluded as data from their questionnaires could not be evaluated. As a result, 3,964 patients were included in the analyses (Table 1) and 3,851 (97.1%) observations contributed to the prevalence estimate of the primary and secondary endpoints

**Table 1:** General demographics, MDRO carriage status and MDRO prevalence in children at hospital admission, AKK Hamburg, Germany, September 2018–May 2019 (n = 3,964)

Characteristics	MDRO negative n = 3,685		MDRO positive n = 166		Missing n = 113	Total n = 3,964		MDRO Prevalence	
	N	%	N	%	n	n	%	% ^a^	95% CI
Sex									
Male	2,046	55.5	95	57.2	54	2,195	55.4	4.43	3.60–5.40
Female	1,639	44.5	71	42.8	59	1,769	44.6	4.15	3.26–5.21
Age (years)									
Mean (SD)	6.4 (5.7)	NA	4.4 (5.4)	NA	NA	6.4 (5.7)	NA	NA	
Median (range: 0.0–18.0)	5.0	NA	2.0	NA	NA	5.0	NA	NA	
0–4 years	1,780	48.3	110	66.3	18	1,908	48.1	5.82	4.81–6.97
5–9 years	651	17.7	19	11.4	29	699	17.6	2.84	1.72–4.39
10–14 years	823	22.3	23	13.9	56	902	22.8	2.69	1.71–4.00
15–18 years	421	11.4	14	8.4	20	455	11.5	3.22	1.77–5.34
Department									
General paediatrics	1,732	47.0	97	58.4	18	1,847	46.6	5.30	4.32–6.43
Paediatric surgery	714	19.4	23	13.9	1	738	18.6	3.12	1.99–4.65
Orthopaedics	566	15.4	12	7.2	89	667	16.8	2.08	1.08–3.60
Trauma surgery	343	9.3	14	8.4	2	359	9.1	3.92	2.16–6.49
Neonatology	125	3.4	4	2.4	0	129	3.3	3.10	0.85–7.75
Otorhinolaryngology	86	2.3	2	1.2	0	88	2.2	2.27	0.28–7.97
Urology	40	1.1	2	1.2	0	42	1.1	4.76	0.58–16.16
Lufthafen	32	0.9	4	2.4	0	36	0.9	11.11	3.11–26.06
Neurosurgery	29	0.8	3	1.8	3	35	0.9	9.38	1.98–25.02
ICU	18	0.5	5	3.0	0	23	0.6	21.74	7.46–43.70
Emergency admission									
No	1,420	38.5	43	25.9	95	1,558	39.3	2.94	2.14–3.94
Yes	2,118	57.5	115	69.3	18	2,251	56.8	5.15	4.27–6.15
Missing information	147	4.0	8	4.8	0	155	3.9	NA	
Transferred from another hospital									
No	3,315	90.0	141	84.9	112	3,568	90.0	4.08	3.44–4.79
Yes	222	6.0	17	10.2	1	240	6.1	7.11	4.20–11.14
Missing information	148	4.0	8	4.8	0	156	3.9	NA	

AKK: Altona Children's Hospital; ICU: intensive care unit; Lufthafen: special unit for children with permanent ventilation; MDRO: multidrug-resistant organisms; NA: not applicable; SD: standard deviation.

^a^ Denominator for calculations = 3,851 patients with information on MDRO status.

Percentages may not add up to 100 because of rounding.

Of the closed-ended questions, 90.2% were answered. Open-ended questions about the country of birth of the child and the parents were answered by 28.4% (n = 1,125) of the parents; of these, 23.7% (n = 266) stated Germany as their birthplace. The participation rate of parents of male children was higher (55.4%) than parents of female children (44.6%). The mean age of participants was 6.4 (SD 5.7) years. Most participants were recruited from general paediatric wards (46.6%), followed by paediatric surgery (18.6%), orthopaedics (16.8%) and trauma surgery (9.1%) (Table 1). Over half of the participants were admitted via the emergency room (56.8%), and the rest were admitted electively (39.3%). Only a few were transferred from other hospitals (6.1%). The three risk factors with the highest positive response rates were (i) having a pet (30.4%), (ii) hospital stay within the previous 12 months (28.8%) and (iii) being breastfed within the previous 12 months (20.7%) (see Supplementary Tables S1–3). Of the 2.8% of participants with a previous positive history of MDRO, more than half had been tested positive for MRGN (1.8%) before the current study. In addition, a refugee status within the previous 12 months (n = 27) and living in a care facility (n = 31) resulted in a similar positive response rate. A higher proportion of participants indicated that they had a disability requiring permanent care (5.4%) or had received antibiotic therapy within the previous 6 months (16.6%). Other characteristics are shown in Table 2 (Supplementary Tables S1–3).

**Table 2:** Risk factors for MDRO colonisation in children, collected at hospital admission, AKK Hamburg, Germany, September 2018–May 2019 (n = 3,964)

Risk factor	MDRO negative n = 3,685		MDRO positive n = 166		Missing n = 113	Total n = 3,964	
	n	%	n	%	n	n	%
**History of MRGN**							
No	3,642	98.8	139	83.7	112	3,893	98.2
Yes	43	1.2	27	16.3	1	71	1.8
Living in a care facility							
No	3,661	99.3	161	97.0	110	3,932	99.2
Yes	23	0.6	5	3.0	3	31	0.8
Missing information	1	0.0	0	0.0	0	1	0.0
**Refugee status within the previous 12 months**							
No	3,656	99.2	161	97.0	113	3,930	99.1
Yes	25	0.7	2	1.2	0	27	0.7
Missing information	4	0.1	3	1.8	0	7	0.2
**Chronic condition requiring permanent care**							
No	3,512	95.3	136	81.9	97	3,745	94.5
Yes	170	4.6	29	17.5	16	215	5.4
Missing information	3	0.1	1	0.6	0	4	0.1
Antibiotic therapy within the previous 6 months							
No	2,961	80.4	104	62.7	89	3,154	79.6
Yes	590	16.0	51	30.7	19	660	16.6
Missing information	134	3.6	11	6.6	5	150	3.8

AKK: Altona Children’s Hospital; MDRO: multidrug-resistant organisms; MRGN: multidrug-resistant Gram-negative bacteria.

Percentages may not add up to 100 because of rounding.

### MDRO prevalence

**Figure 1:**
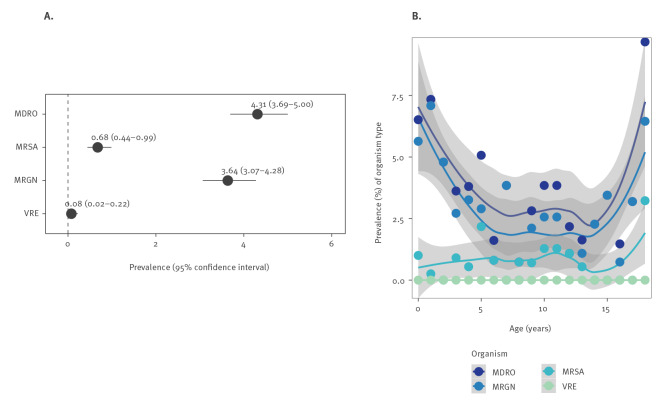
Overall MDRO prevalence (A) and age-specific MDRO prevalence (B) in children at hospital admission, AKK Hamburg, Germany, September 2018–May 2019 (n = 3,851)^a^

AKK: Altona Children’s Hospital; LOESS: locally weighted scatterplot smoothing; MDRO: multidrug-resistant organisms; MRGN: multidrug-resistant Gram-negative bacteria; MRSA: meticillin-resistant *Staphylococcus aureus*; VRE: vancomycin-resistant enterococcus.

^a^ Results for MDRO and MRGN are based on 3,851 observations, MRSA on 3,849 and VRE on 3,908.

^b^ LOESS estimate for smoothed trend.

The prevalence of MDRO carriage at admission was 4.31% (95% CI: 3.69–5.00): MRGN prevalence was the highest (3.64%, 95% CI: 3.07–4.28), MRSA prevalence was lower (0.68%, 95% CI: 0.44–0.99) and VRE was the lowest (0.08%, 95% CI: 0.02–0.22) (Figure 1A).

### Comparison of screening algorithms at admission

Based on the AKK established admission screening using 17 risk factors, 109 (65.7%) of 166 MDRO cases would have been detected, as they reported at least one risk factor resulting in MDRO screening (Table 3). Based on the extended screening of 25 risk factors, as implemented in the current study (see methods), 143 (86.1%) MDRO cases would have been detected. Comparing both screening algorithms regarding their diagnostic performance, sensitivity (86.13%, 95% CI: 80.89–91.40) and specificity (73.54%, 95% CI: 72.12–74.97) were higher for the extended risk-factor-based screening compared with the established screening method.

**Table 3:** Diagnostic performance, detected MDRO cases and MDRO prevalence in children by MDRO screening algorithm at hospital admission, AKK Hamburg, Germany, September 2018–May 2019 (n = 418 detected MDRO cases, results are based on 3,851 observations)

Screening algorithm	Sensitivity		Specificity		Detected MDRO cases	Estimated MDRO prevalence
	%	95% CI	%	95% CI	n	%
Established (17 factors)	65.66	58.44–72.89	45.10	43.50–46.71	109	2.75
Extended (25 factors)	86.14	80.89–91.40	73.54	72.12–74.97	143	3.61
Universal	NA		NA		166	4.31

AKK: Altona Children’s Hospital; CI: confidence interval; MDRO: multidrug-resistant organisms; NA: not applicable.

Sensitivity and specificity were assessed using the original and extended screening survey along with 95% Wald CI.

### MDRO species and frequency

Of the 30 participants with MRSA carriage, 21 were detected by nasal/throat screening; of the 153 participants with MRGN carriage, 130 (85.0%) were detected by inguinal/anal swabs (Table 4). of the 2MRGN (85 pathogen detection) was the highest MRGN colonisation, followed by 3MRGN (66 pathogen detection) and 4MRGN (1 participant two pathogen detection). The most frequent MRGN species were *E. coli* and *Enterobacter* spp*.* The 4MRGN were *K. pneumoniae* and *Acinetobacter baumannii*, both detected from nasal/throat swab. Carbapenemase of the OXA-48 group was detected in 4MRGN *K. pneumoniae,* and carbapenemase of the OXA-23 group was detected in 4MRGN *A. baumannii*. The child with detected 4MRGN had a chronic condition requiring permanent care and implanted medical devices and did not have refugee status. All three participants with VRE carriage swabbed positive for *Enterococcus faecium.* More than one different MDRO in the same patient was determined for three participants (0.08%) – one with MRSA and 2MRGN, one with MRSA and 3MRGN and one with two different 4MRGN.

**Table 4:** MDRO species frequencies in children by swab location at hospital admission, AKK Hamburg, Germany, September 2018–May 2019 (n = 3,964 children)

MDRO species	Swab location				Total swabs n = 7,928	
	Nasal/throat n = 3,964		Inguinal/anal n = 3,964			
	n	%	n	%	n	%
MRSA	21	0.53	9	0.23	30	0.38
MRGN	23	0.58	130	3.28	153	1.92
*Escherichia coli*	9	0.23	99	2.50	108	1.36
*Enterobacter species*	1	0.03	8	0.20	9	0.11
*Klebsiella pneumoniae*	2	0.05	5	0.13	7	0.09
*Acinetobacter baumannii*	2	0.05	5	0.13	7	0.09
*Citrobacter species*	0	0.00	3	0.08	3	0.04
*Serratia species*	0	0.00	1	0.03	1	0.01
*Proteus species*	0	0.00	1	0.03	1	0.01
*Pseudomonas aeruginosa*	1	0.03	0	0.00	1	0.01
Other	8	0.20	8	0.20	16	0.20
Not available	4	0.10	111	2.80	115	1.45
No MRSA or MRGN detected	3,893	98.21	3,584	90.41	7,477	94.31

AKK: Altona Children's Hospital; MDRO: multidrug-resistant organisms; MRGN: multidrug-resistant Gram-negative bacteria; MRSA: meticillin-resistant *Staphylococcus aureus*.

### Sex-, age- and department-specific MDRO prevalence

The prevalence of MDRO was similar in females (4.01%) and males (4.33%). Estimates of age-related prevalence revealed that MDRO and MRGN prevalence tended to be higher in the first years of life but also in adolescence (Figure 1B). MRSA carriage prevalence was constant over all ages (Figure 1B). The highest prevalence of MDRO carriage was observed in patients in the ICU (21.74%), the Lufthafen (the special unit for children on permanent ventilation) (11.11%) and the neurosurgical ward (9.38%) (Table 1).

### Associations between risk factors and MDRO occurrence

**Figure 2:**
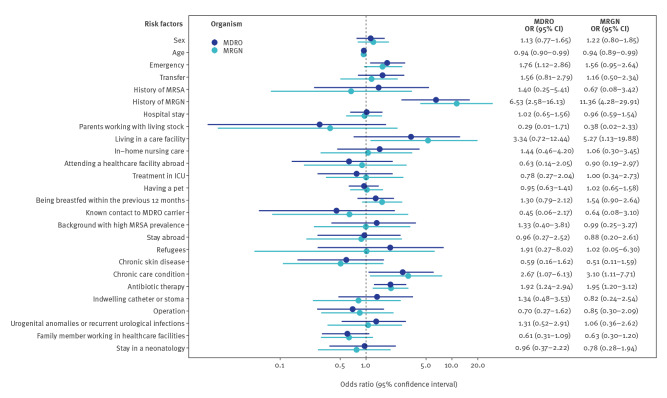
Associations between risk factors and MDRO and MRGN in children at hospital admission, AKK Hamburg, Germany, September 2018–May 2019 (n = 3,017)

AKK: Altona Children's Hospital; ICU: intensive care unit; MDRO: multidrug-resistant organisms; MRGN: multidrug-resistant Gram-negative bacteria; MRSA: meticillin-resistant *Staphylococcus aureus*.

The most important risk factor for MDRO was MRGN history. An OR of 6.53 (95% CI: 2.58–16.13) indicated that the odds for MDRO occurrence in patients with previously reported MRGN was more than six times higher than for a patient without a previous MRGN history (Figure 2). Other clinically relevant associations with MDRO were living in a care facility (OR 3.34; 95% CI: 0.72–12.44), refugee status within the previous 12 months (OR: 1.91; 95% CI: 0.27–8.02), chronic condition requiring permanent care (OR: 2.67; 95% CI: 1.07–6.13) and antibiotic therapy within the previous 6 months (OR: 1.92; 95% CI: 1.24–2.94). Similar risk factors were also identified for MRGN colonisation, although more pronounced associations were identified for MRGN history (OR: 11.36; 95% CI: 4.28–29.91), living in a care facility (OR: 5.27; 95% CI: 1.13–19.88) and chronic care condition (OR: 3.10; 95% CI: 1.11–7.71) (Figure 2).

Odds ratio estimates were derived from logistic regression models as were 95% confidence intervals for MDRO and MRGN colonisation.

Results based on 3,017 complete observations.

In the figure, the risk factors are briefly summarized as keywords.

## Discussion

In this study, we investigated the prevalence of MDRO carriage at admission to AKK, a major paediatric hospital in Hamburg, for scheduled or emergency admissions over 9 months.

We found an overall prevalence of MDRO carriage in children of 4.31% (95% CI: 3.69–5.00) at admission to hospital. This prevalence is higher than reported MDRO prevalence estimates in children in European studies up to 2014 [15,16], and similar to recent Danish and US studies examining paediatric and adult populations [10,13]. Previous European studies reported an MDRO prevalence in adults ranging from 3.04% [23], 3.46% [9], 5.2% (95% CI: 4.6–5.8 [10]), 7.2% [12] up to 10% [20].

The MRGN prevalence of 3.64% (95% CI: 3.07–4.28) in this study was higher than the observed prevalence in another German study examining adults (1.65%) [9] and similar to a recent Dutch study examining adults (5.0%, 95% CI: 3.4–6.6) [2]. Recent European studies have found an increasing ESBL carriage prevalence in preschool children, from 3.5% (95% CI: 2.5–4.8%) [13] to 16.8% [30], much greater than the MRSA carriage prevalence in children, from 1.2 [13] to 2.7% (95% CI: 2.2–3.1) [14]. This study confirmed a low prevalence of MRSA carriage of 0.68% (95% CI: 0.44–0.99) at hospital admission.

VRE had a reported low prevalence of 0.25% (95% CI: 0.13–0.37) [9] to 0.4% (95% CI: 0.3–0.6) [10] in previous German studies examining adults [9,23] and was only detected in three patients in this study (0.08%, 95% CI: 0.02–0.22).

The highest MDRO and MRGN carriage prevalence was observed in children aged 1–5 years and those 18 years of age. Possible reasons for the increased MRGN prevalence in the first years of life are physical contacts, crowding, difficulty in complying with basic hygiene routines, high likelihood for exposure to antimicrobial drugs, household-transmission and attendance at preschools and day care centres [22,30,31]. The increase in MDRO prevalence for 18-year-olds could be because most patients hospitalised at this age at a paediatric hospital have chronic paediatric diseases. That is, these patients may have had more contact with healthcare facilities in the past.

The difference in MDRO prevalence in Europe can be explained by the use of different MDRO definitions and by the evidence of a north-to-south and west-to-east gradient according to the latest surveillance report from the European Centre for Disease Prevention and Control (ECDC) and recent European studies [11,32,33]. As northern and western European countries seem to show a similar picture regarding MDRO occurrence, the results of this study are similar to the results of Danish and Dutch studies in adults [2,10]. Therefore, the results of this study might be primarily applicable to northwestern European regions; further Europe-wide children’s studies are required for confirmation of these findings.

The AKK is equipped with a hygiene commission consisting of four doctors and three trained hygiene staff who meet once a month to discuss hygiene complications and review existing screening forms against possible changes in KRINKO recommendations. To date, screening procedures at the AKK require nasal/throat or inguinal/anal swabs if patients reported one of 17 risk factors for MRSA or MRGN/VRE colonisation and nasal/throat swabs from all intensive care and neonatology patients. Isolation is performed after receiving a positive result, unless a colonisation was known beforehand. In this study, we extended our locally established questionnaire by adding additional eight risk factors and further subsequent questions, as there is no common agreement on relevant risk factors [12].

Similar to findings by Hamprecht et al. in 2016, the following risk factors for MDRO colonisation were identified: MRGN history, chronic condition requiring permanent care, antibiotic therapy within the previous 6 months, living in a care facility and refugee status within the previous 12 months [14]. Relevant risk factors for MRGN colonisation were the same. Because of the low number of cases, we could not provide relevant risk factors for MRSA and VRE colonisation.

The locally established screening at the AKK, which is similar to national and international standards, would have missed several MDRO cases (57 of 166) (Table 3). By adding eight risk factors, we were able to improve diagnostic performance (sensitivity: 86.13%, 95% CI: 80.89–91.40; specificity: 73.54%, 95% CI: 72.12–74.97). As screening all in-patients is not reasonable because of low prevalence and high screening costs, the best option might be an extended risk-factor-based admission screening system, as this increases the detection rate of MDRO carriers, secures adequate therapies and reduces health costs [12,34]. Moreover, it is also possible to use the extended risk-factor-based admission screening routinely at emergency units as the majority of the questions are relevant to the patient’s medical history, which would need to be reported in any case. As a consequence of this study’s findings, the extended screening system is now being established at the AKK.

When classifying MRGN cases according to antibiotic resistance, we primarily detected 2MRGN, followed by 3MRGN and 4MRGN. The detected carbapenemase of the OXA-48 group in 4MRGN *K. pneumoniae* and of the OXA-23 group in 4MRGN *A. baumannii* corresponds to the predominance in Europe [18] and Germany. According to KRINKO guidelines, 2MRGN colonisation is relevant in paediatric ICUs and other risk zones, such as neonatology units because of mandatory isolation and their same therapeutic status as 3MRGN [22]. Therefore, we also had the highest MDRO prevalence in our clinic risk zones, the ICU and the Lufthafen (Table 1).

Among the risk factors identified as having the highest positive response rate, being breastfed within the previous 12 months was identified in 20.7% of children with MDRO. Interestingly, Saporito et al. saw in 2020, a correlation between high MRGN colonisation and being breastfed (including breast milk feeding from bottles) [35] and recommend further evaluation of the handling and storage of the expressed breast milk before administration [35].

Lastly, 2MRGN colonisation must be viewed as a pre-stage to 3MRGN and 4MRGN stages. It is likely that the concerned participants in this study will develop bacterial infections that require antibiotic treatment, resulting in the development of further resistances within their positive MDRO colonisation status. It is known that positive MDRO colonisation can disappear for several years and return under selection pressure, such as exposure to antibiotics or travel to Africa and Asia [7,19,36,37]. Moreover, MRSA eradication treatments are available, but currently no therapeutic recommendations for MRGN colonisation exist. In line with our findings, other studies have also demonstrated only few *E. coli* or *Enterobacter* spp. in throat swabs. The clinical significance of *E.coli* (nasal/throat) as a pathogen is not clearly understood in paediatric patients [38], whereas sepsis caused by *Enterobacter* spp. and *Escherichia* spp. is associated with a high risk of mortality in the very low birth weight paediatric population [39].

Overall, this study had a high response rate: of the 4,092 patients recruited, 3,851 (97.1%) were included in our analyses. Although we generated high quality data, some questionnaires were incomplete, especially on days with a high patient influx at the clinic and thus some important risk factors, including patient/family member with treatment in an ICU, antibiotic treatments, parents working in healthcare facilities and country of birth, may have been missed. Because of the low number of cases or missing answers, five factors (country of birth of the child and parents, department, VRE history, dialysis and treatment of a family member in an ICU) and the 18 sub-questions could not be included in any of the logistic regression models. Data obtained from adolescent patients were generally more incomplete than from their younger counterparts as some adolescents refused anal swabs. This situation generated higher numbers of unknown anal and inguinal colonisation events, probably resulting in several missed MDRO cases. As previous studies have shown, taking a rectal swab has a higher certitude of detection than anal swabs. Because we already had a lower response for adolescence anal swabs, we would expect an even lower compliance if rectal swabs had been required. In addition, our results could have been influenced by the low number of participants who had refugee status (n = 27) [1] or who lived in a care facility (n = 31) [2], but it seems realistic to assume that the refugees are a minority among children being admitted at AKK. If the proportion of these had been higher, we would probably have had higher MDRO prevalence rates as other studies in Germany have shown among refugees and residents of care facilities [9,21,23,37,40].

Unfortunately, due to financial limitations, it was not possible to perform MDRO subtyping or to recruit participants over the entire year. Therefore, with regard to the described seasonal accumulations of, for example, ESBL occurrence in August and September, a year-round study would probably have shown an even higher MRGN prevalence [36].

Screening of parents was not performed and their medical history did not explicitly include questions about colonisation even though, as reported by van den Bunt et al. in 2016, parents of MDRO-positive children might also be carriers [31]. In any case, the source of parental colonisation would most likely be unknown. However, MDRO and especially ESBL transmission via contaminated items such as food and/or cloth (e.g., kitchen towels) has also been described as an MDRO risk factor [2,12].

### Conclusion

Children and adolescents admitted to the AKK most likely represent the paediatric populations of Germany. This study has shown that the prevalence of colonisation of children with MRGN bacteria is higher than MRSA and VRE occurrence and that existing screening procedures can be optimised to decrease undetected MDRO colonisation events. Currently, the best option to detect MDRO in children from northern and western parts in Europe (or comparable low prevalence settings) might be an extended risk-factor-based admission screening system that considers healthcare-related risk factors. Therefore, there is a high need for further European studies on the modification of admission screening algorithms with a focus on MRGN in preschool children as this was the age group with the highest prevalence in our study.

## Supplementary Data

287000 Supplementary Data
