# Supplementary material for: High prevalence of multidrug-resistant Gram-negative bacteria carriage in children screened prospectively for multidrug resistant organisms at admission to a paediatric hospital, Hamburg, Germany, September 2018 to May 2019
Source: Euro Surveill. 2022 Apr 14;27(15):2001567. doi: 10.2807/1560-7917.ES.2022.27.15.2001567 (PMC9012092; doi:10.2807/1560-7917.ES.2022.27.15.2001567)
Supplement: Supplementary Data [file 20-01567_KOENIGS_SupplementaryData.pdf]

Supplementary Data

MDRO risk factors and MDRO carriage in children, collected at time of hospital admission, AKK Hamburg, Germany, 2018-2019

This supplementary material is hosted by Eurosurveillance as supporting information alongside the article [High prevalence of multi-drug-resistant gram-negative bacteria carriage in children at time of hospital admission], on behalf of the authors, who remain responsible for the accuracy and appropriateness of the content. The same standards for ethics, copyright, attributions and permissions as for the article apply. Supplements are not edited by Eurosurveillance and the journal is not responsible for the maintenance of any links or email addresses provided therein.

Table S1. MDRO risk factors and MDRO carriage in children, collected at time of hospital admission, AKK Hamburg, Germany, 2018-2019 – part 1

N	Risk factor	MDRO negative n = 3685		MDRO positive n = 166		Missing n = 113	Total n = 3964	
		(n)	(%)	(n)	(%)	(n)	(n)	(%)
1.	**History of MRSA**							
	No	3655	99.2	159	95.8	111	3925	99.0
	Yes	30	0.8	7	4.2	2	39	1.0
1.1.	**Localization of MRSA**							
	Nasal/throat	16	N/A	4	N/A	0	20	N/A
	Wound	2	N/A	1	N/A	0	3	N/A
	Other	2	N/A	0	N/A	0	2	N/A
	Missing	3665	99.5	161	97.0	113	3939	99.4
2.	**History of MRGN**							
	No	3642	98.8	139	83.7	112	3893	98.2
	Yes	43	1.2	27	16.3	1	71	1.8
2.1.	**Localization of MRGN**							
	Anal/rectal/stool	3642	98.8	139	83.7	112	3893	98.2
	Wound	26	N/A	16	N/A	0	42	N/A
	Urine	1	N/A	3	N/A	1	5	N/A
	Other	11	N/A	4	N/A	0	15	N/A
	Missing	5	0.1	4	2.4	0	9	0.2
3.	**History of VRE**							
	No	3684	100	166	100	113	3963	100
	Yes	1	0.0	0	0.0	0	1	0.0
3.1.	**Localization of VRE**							
	Anal/rectal/stool	1	N/A	0	N/A	0	1	N/A
	Missing	3684	100.0	166	100	113	3963	100
4.	**Hospital stay within the previous 12 months**							
	No	2656	72.1	99	59.6	59	2814	71.0
	Yes	1022	27.7	66	39.8	54	1142	28.8
	Missing	7	0.2	1	0.6	0	8	0.2
4.1.	**Number**							
	1	679	18.4	30	18.1	27	736	18.6
	>1	242	6.6	28	16.9	11	281	7.1
	Missing	2764	75.0	108	65.1	75	2947	74.3
4.2.	**Duration (in days)**							
	0–3	412	11.2	24	14.5	24	460	11.6
	4–5	190	5.2	5	3.0	8	203	5.1
	6–10	131	3.6	11	6.6	7	149	3.8
	11–20	65	1.8	5	3.0	2	72	1.8
	>20	122	3.3	11	6.6	5	138	3.5
	Missing	2765	75.0	110	66.3	67	2942	74.2
5.	**Treatment in dialysis department**							
	No	3681	99.9	166	100	112	3959	99.9
	Yes	4	0.1	0	0.0	1	5	0.1
6.	**Parents working with living stock**							
	No	3507	95.2	158	95.2	113	3778	95.3
	Yes	48	1.3	1	0.6	0	49	1.2
	Missing	130	3.5	7	4.2	0	137	3.5
7.	**Living in a care facility**							
	No	3661	99.3	161	97.0	110	3932	99.2
	Yes	23	0.6	5	3.0	3	31	0.8
	Missing	1	0.0	0	0.0	0	1	0.0
8.	**In-home nursing care**							
	No	3625	98.4	151	91.0	111	3887	98.1
	Yes	60	1.6	14	8.4	2	76	1.9
	Missing	0	0.0	1	0.6	0	1	0.0
9.	**Attending a healthcare facility abroad within the previous 12 months**							
	No	3601	97.7	162	97.6	111	3874	97.7
	Yes	84	2.3	4	2.4	2	90	2.3
9.1.	**Number**							
	1	47	N/A	4	N/A	2	53	N/A
	>1	10	N/A	0	N/A	0	10	N/A
	Missing	3628	98.5	162	97.6	111	3901	98.4
9.2.	**Duration (in days)**							
	0–3	37	N/A	2	N/A	1	40	N/A
	4–5	8	N/A	0	N/A	0	8	N/A
	6–10	5	N/A	1	N/A	1	7	N/A
	11–20	2	N/A	1	N/A	0	3	N/A
	>20	2	N/A	0	N/A	0	2	N/A
	Missing	3631	98.5	162	97.6	111	3904	98.5

Abbreviations: AKK, Altona Children's Hospital; ICU, intensive care unit; MDRO, multi-drug-resistant organisms; MRGN, multi-drug-resistant gram-negative bacteria; MRSA, methicillin-resistant Staphylococcus aureus; n., number; N/A, not applicable; PEG, percutaneous endoscopic gastrostomy; VRE, vancomycin-resistant enterococcus.
Percentages may not total 100 due to rounding. Missing, questions not answered. Results are based on 3851 observations.

Table S2. MDRO risk factors and MDRO carriage in children, collected at time of hospital admission, AKK Hamburg, Germany, 2018-2019 -part 2

N	Risk factor	MDRO negative n = 3685		MDRO positive n = 166		Missing n = 113	Total n = 3964	
		(n)	(%)	(n)	(%)	(n)	(n)	(%)
**9.**	**Attending a healthcare facility abroad within the previous 12 months**							
**9.3.**	**Continent**							
	Africa	5	N/A	0	N/A	0	5	N/A
	Asia	5	N/A	1	N/A	0	6	N/A
	Europe	42	N/A	1	N/A	2	45	N/A
	Eastern Europe	9	N/A	1	N/A	0	10	N/A
	Southern Europe	9	N/A	1	N/A	0	10	N/A
	North America	3	N/A	0	N/A	0	3	N/A
	South America	1	N/A	0	N/A	0	1	N/A
	Middle East	5	N/A	0	N/A	0	5	N/A
	Australia/New Zealand	2	N/A	0	N/A	0	2	N/A
	Missing	3604	97.8	162	97.6	111	3877	97.8
**10.**	**Treatment in ICU for more than seven days within the previous 12 months**							
	No	3580	97.2	151	91.0	113	3844	97.0
	Yes	100	2.7	14	8.4	0	114	2.9
	Missing	5	0.1	1	0.6	0	6	0.2
**11.**	**Contact with a treated family member in ICU for more than seven days within the previous 12 months**							
	No	3366	91.3	148	89.2	113	3627	91.5
	Yes	52	1.4	0	0.0	0	52	1.3
	Missing	267	7.2	18	10.8	0	285	7.2
**12.**	**Having a pet**							
	No	2303	62.5	104	62.7	71	2478	62.5
	Yes	1123	30.5	50	30.1	33	1206	30.4
	Missing	259	7.0	12	7.2	9	280	7.1
**13.**	**Breastfeeding during the last 12 months**							
	No	2894	78.5	113	68.1	105	3112	78.5
	Yes	760	20.6	51	30.7	8	819	20.7
	Missing	31	0.8	2	1.2	0	33	0.8
**14.**	**Known contact with a MDRO carrier**							
	No	3659	99.3	164	98.8	113	3936	99.3
	Yes	26	0.7	2	1.2	0	28	0.7
**15.**	**Background with known high MRSA prevalence**							
	No	3614	98.1	158	95.2	109	3881	97.9
	Yes	71	1.9	8	4.8	4	83	2.1
**16.**	**Abroad for > six weeks within the previous six months**							
	No	3554	96.4	161	97.0	109	3824	96.5
	Yes	113	3.1	4	2.4	3	120	3.0
	Missing	18	0.5	1	0.6	1	20	0.5
**17.**	**Refugee status within the previous 12 months**							
	No	3656	99.2	161	97.0	113	3930	99.1
	Yes	25	0.7	2	1.2	0	27	0.7
	Missing	4	0.1	3	1.8	0	7	0.2
**17.1**	**Moving in within the previous three months**							
	No	3674	99.7	163	98.2	113	3950	99.6
	Yes	6	0.2	0	0.0	0	6	0.2
	Missing	5	0.1	3	1.8	0	8	0.2
**17.2**	**Staying at a community center within the previous three months**							
	No	3668	99.5	162	97.6	113	3943	99.5
	Yes	11	0.3	1	0.6	0	12	0.3
	Missing	6	0.2	3	1.8	0	9	0.2
**18.**	**Chronic skin disease**							
	No	3529	95.8	158	95.2	110	3797	95.8
	Yes	155	4.2	8	4.8	3	166	4.2
	Missing	1	0.0	0	0.0	0	1	0.0
**18.1.**	**Type**							
	Chronic wound	8	N/A	2	N/A	0	10	N/A
	Ulcus	3	N/A	0	N/A	0	3	N/A
	Deep soft tissue infection	7	N/A	1	N/A	0	8	N/A
	Neurodermatitis	102	2.8	4	2.4	2	108	2.7
	Other	29	N/A	1	N/A	1	31	N/A
	Missing	3536	96.0	158	95.2	110	3804	96.0
**19.**	**Chronic condition requiring permanent care**							
	No	3512	95.3	136	81.9	97	3745	94.5
	Yes	170	4.6	29	17.5	16	215	5.4
	Missing	3	0.1	1	0.6	0	4	0.1

Abbreviations: AKK, Altona Children's Hospital; ICU, intensive care unit; MDRO, multi-drug-resistant organisms; MRGN, multi-drug-resistant gram-negative bacteria; MRSA, methicillin-resistant Staphylococcus aureus; n., number; N/A, not applicable; PEG, percutaneous endoscopic gastrostomy; VRE, vancomycin-resistant enterococcus.
Percentages may not total 100 due to rounding. Missing, questions not answered. Results are based on 3851 observations.

Table S3. MDRO risk factors and MDRO carriage in children, collected at time of hospital admission, AKK Hamburg, Germany, 2018-2019 -part 3

N	Risk factor	MDRO negative n = 3685		MDRO positive n = 166		Missing n = 113	Total n = 3964	
		(n)	(%)	(n)	(%)	(n)	(n)	(%)
**20.**	**Antibiotic therapy within the previous 6 months**							
	No	2961	80.4	104	62.7	89	3154	79.6
	Yes	590	16.0	51	30.7	19	660	16.6
	Missing	134	3.6	11	6.6	5	150	3.8
**20.1.**	**Oral continuous prophylaxis**							
	No	3498	94.9	151	91.0	105	3754	94.7
	Yes	36	N/A	4	N/A	3	43	N/A
	Missing	151	4.1	11	6.6	5	167	4.2
**20.2.**	**Number of oral therapies**							
	None	3047	82.7	113	68.1	95	3255	82.1
	1	314	8.5	25	15.1	9	348	8.8
	2-3	102	2.8	10	6.0	0	112	2.8
	>3	40	N/A	4	N/A	0	44	N/A
	Missing	182	4.9	14	8.4	9	205	5.2
**20.3.**	**Number of intravenous therapies**							
	None	3406	92.4	139	83.7	101	3646	92.0
	1	56	1.5	6	3.6	1	63	1.6
	2-3	25	N/A	2	N/A	0	27	N/A
	>3	15	N/A	5	N/A	2	22	N/A
	Missing	183	5.0	14	8.4	9	206	5.2
**20.4.**	**Duration of treatment (days)**							
	≤7	215	5.8	17	10.2	6	238	6.0
	>7	164	4.5	18	10.8	6	188	4.7
	Missing	3306	89.7	131	78.9	101	3538	89.3
**21.**	**Indwelling catheter or stoma**							
	No	3565	96.7	145	87.3	108	3818	96.3
	Yes	120	3.3	21	12.7	5	146	3.7
**21.1.**	**Type**							
	Gastrostomy/PEG/Anus praeter	33	N/A	8	N/A	1	42	N/A
	Tracheostomy	13	N/A	5	N/A	0	18	N/A
	Central venous catheter/ Broviac catheter/ Port	10	N/A	1	N/A	2	13	N/A
	Transurethral/ suprapubic catheter	3	N/A	0	N/A	0	3	N/A
	Baclofen pump	3	N/A	3	N/A	0	6	N/A
	Ventriculoperiteoneal/ventriculo-atrial shunt	23	N/A	3	N/A	2	28	N/A
	Insulin pump	31	N/A	1	N/A	0	32	N/A
	Missing	3569	96.9	145	87.3	108	3822	96.4
**22.**	**Major surgery within the previous 12 months**							
	No	3480	94.4	154	92.8	83	3717	93.8
	Yes	199	5.4	12	7.2	25	236	6.0
	Missing	6	0.2	0	0.0	5	11	0.3
**22.1.**	**Localization**							
	Vessel	9	N/A	0	N/A	0	9	N/A
	Heart	4	N/A	2	N/A	1	7	N/A
	Bone	117	3.2	3	1.8	23	143	3.6
	Abdomen	50	N/A	7	N/A	1	58	N/A
	Missing	3505	95.1	154	92.8	88	3747	94.5
**23.**	**Urogenital anomalies or recurrent urological infections**							
	No	3564	96.7	157	94.6	110	3831	96.6
	Yes	119	3.2	9	5.4	2	130	3.3
	Missing	2	0.1	0	0.0	1	3	0.1
**24.**	**Family member working in healthcare facilities with regular patient contact**							
	No	2809	76.2	136	81.9	91	3036	76.6
	Yes	507	13.8	15	9.0	18	540	13.6
	Missing	369	10.0	15	9.0	4	388	9.8
**24.1.**	**Place**							
	Hospital	158	4.3	6	3.6	8	172	4.3
	ICU	17	N/A	0	N/A	0	17	N/A
	Doctor`s office	129	3.5	4	2.4	4	137	3.5
	Nursing home	83	2.3	3	1.8	4	90	2.3
	Other	81	2.2	1	0.6	1	83	2.1
	Missing	3217	87.3	152	91.6	96	3465	87.4
**25.**	**Stay at a neonatology unit for more than seven days within the previous 12 months**							
	No	3552	96.4	149	89.8	113	3814	96.2
	Yes	131	3.6	16	9.6	0	147	3.7
	Missing	2	0.1	1	0.6	0	3	0.1

Abbreviations: AKK, Altona Children's Hospital; ICU, intensive care unit; MDRO, multi-drug-resistant organisms; MRGN, multi-drug-resistant gram-negative bacteria; MRSA, methicillin-resistant Staphylococcus aureus; n., number; N/A, not applicable; PEG, percutaneous endoscopic gastrostomy; VRE, vancomycin-resistant enterococcus.
Percentages may not total 100 due to rounding. Missing, questions not answered. Results are based on 3851 observations.
